# First we feast: a narrative review of postprandial assessment of left ventricular outflow tract obstruction in hypertrophic cardiomyopathy

**DOI:** 10.1186/s44156-026-00135-1

**Published:** 2026-09-01

**Authors:** Mahesh Chandrasekhar, Carolyn Park, Pamela Burgess, Richard Brandon Stacey

**Affiliations:** 1 Cone Heart and Vascular Institute, Greensboro, NC USA; 2 Atrium Wake Forest Baptist Medical Center, Winston-Salem, NC USA

**Keywords:** Hypertrophic cardiomyopathy, Left ventricular outflow tract obstruction, Postprandial, Echocardiography, Gradient, Provocative testing

## Abstract

**Background:**

Dynamic left ventricular outflow tract (LVOT) obstruction is a common and consequential feature of hypertrophic cardiomyopathy (HCM), present in a majority of patients at rest or with provocation, and it directly influences symptom burden and eligibility for therapy. When obstruction is absent at rest it can be provoked by physiologic stimuli, including the ingestion of a meal, a phenomenon patients have reported for decades. Whether postprandial assessment improves detection of obstructive physiology over conventional provocative testing has not been systematically examined.

**Methods:**

We searched PubMed/MEDLINE, Embase, the Cochrane Library, and Web of Science from inception through June 2025. Study quality was assessed with the Newcastle-Ottawa Scale. Because of substantial heterogeneity in study protocols and outcome definitions, we performed a narrative synthesis rather than a meta-analysis.

**Results:**

Seven studies met inclusion criteria, published between 1991 and 2024 across three countries. Across these studies, postprandial assessment consistently provoked higher LVOT gradients than fasting measurement and identified obstructive physiology in patients with no obstruction on conventional testing. In the most rigorous comparative study, postprandial echocardiography identified gradients ≥ 30 mmHg in 74% of patients with no resting obstruction, compared with 23% by Valsalva maneuver (*p* < 0.001). In the largest cohort (*n* = 252), postprandial testing identified gradients ≥ 50 mmHg in an additional 35.7% of patients who did not reach that threshold under routine conditions. Invasive hemodynamic data provide a mechanistic basis: meal ingestion reduced systemic vascular resistance and raised cardiac filling pressures, conditions that favor dynamic obstruction.

**Conclusions:**

Postprandial LVOT assessment identifies obstructive physiology that conventional provocative maneuvers miss. As approved medical therapies targeting LVOT obstruction reshape HCM management, accurate identification of the obstructive phenotype carries direct therapeutic consequences. Standardized multicenter validation is needed, but existing evidence supports consideration of postprandial testing in symptomatic patients who remain without obstruction on conventional assessment.

## Introduction

Hypertrophic cardiomyopathy (HCM) is the most common inherited cardiac disorder, with an estimated prevalence of approximately 1 in 500 in the general population [1]. Dynamic left ventricular outflow tract (LVOT) obstruction, defined as a peak instantaneous gradient ≥ 30 mmHg, is present in roughly two-thirds of patients either at rest or with provocation and is a major determinant of symptoms and a central factor in treatment selection [2–4]. Approximately one-third of patients have obstruction at rest, and a further third develop it only under provocative conditions, while the remainder do not obstruct under any condition [5]. This distinction matters clinically: obstructive physiology determines eligibility for septal reduction therapy and, more recently, for cardiac myosin inhibitor (CMI) therapy [6, 7].

Current guidelines recommend provocative testing with the Valsalva maneuver, exercise echocardiography, or pharmacologic agents when resting obstruction is absent [6, 8]. Yet a substantial proportion of patients report exacerbation of symptoms specifically after meals, an observation documented in the clinical literature for more than three decades [9, 10]. Eating lowers systemic vascular resistance and afterload while raising adrenergic tone, changes that promote systolic anterior motion (SAM) of the mitral valve and dynamic obstruction [11]. Despite this physiologic rationale and the longstanding clinical recognition of postprandial symptoms, postprandial assessment has not been incorporated into standard HCM evaluation, and the available evidence has not been synthesized.

This review examines the available evidence for postprandial LVOT assessment in HCM. We compare its diagnostic yield with that of conventional provocative maneuvers and review the hemodynamic mechanisms that underlie it. We also consider what these findings mean now that accurate identification of obstruction governs access to targeted pharmacologic therapy.

## Methods

### Protocol and reporting

This was conducted as a narrative synthesis. We initially intended a PRISMA-style systematic review with meta-analysis, but the small number of eligible studies and the heterogeneity of their protocols and outcome measures did not support quantitative pooling. We therefore report a narrative synthesis and used the PRISMA framework to guide the literature search and study selection. The review was not prospectively registered.

For the purposes of this review, postprandial assessment was defined as echocardiographic measurement of the LVOT gradient after ingestion of a meal, within a defined post-meal window. Assessment could be performed at rest, with provocative maneuvers, or with exercise. This definition is consistent with the approach used in contemporary clinical practice and in the largest published cohort [12].

### Search strategy

We searched PubMed/MEDLINE, Embase, the Cochrane Library, and Web of Science from inception through June 2025. The search combined Medical Subject Headings and free-text terms as follows: (“hypertrophic cardiomyopathy” OR “HCM” OR “hypertrophic obstructive cardiomyopathy”) AND (“postprandial” OR “post-prandial” OR “after meal” OR “food intake” OR “meal” OR “food challenge”) AND (“left ventricular outflow tract” OR “LVOT” OR “gradient” OR “obstruction” OR “provocative echocardiography” OR “dynamic obstruction”). We hand-searched the reference lists of included studies for additional citations.

### Study selection

Studies were eligible if they enrolled patients with a confirmed diagnosis of HCM, compared LVOT gradient or velocity between fasting and postprandial states, reported quantitative measurements, included at least 10 patients, and were published as full peer-reviewed manuscripts. We excluded case reports, small case series, review articles, editorials, conference abstracts without full data, and studies limited to patients younger than 18 years. Two reviewers (M.C. and R.B.S.) screened titles, abstracts, and full texts against these criteria and identified the same set of eligible studies.

### Data extraction and quality assessment

We extracted study design, patient demographics, meal protocol, echocardiographic methodology, body position, fasting and postprandial LVOT measurements, comparator maneuvers, and clinical outcomes. Methodological quality was assessed with the Newcastle-Ottawa Scale, with a score of 7 or higher considered high quality.

### Synthesis

We did not perform quantitative pooling because of clinical and methodological heterogeneity across studies. Meal protocols ranged from a standardized 740 kcal mixed meal to patient-directed eating, assessment timing ranged from 15 to 60 min after ingestion, patient positioning ranged from supine rest to standing after exercise, and outcomes were variously defined by LVOT velocity, absolute gradient thresholds of 30 or 50 mmHg, or the proportion of patients achieving obstruction. We synthesized findings narratively, reporting point estimates and confidence intervals for individual studies where calculable. Because fewer than 10 studies were included, we did not formally assess publication bias, although the predominance of positive findings in a small and specialized literature raises the possibility that it is present.

## Results

### Study selection

The search identified 247 records, of which seven met inclusion criteria after removal of duplicates and screening by title, abstract, and full text. The most common reasons for exclusion at full text were absence of quantitative gradient data, pediatric-only populations, and insufficient sample size.

### Study characteristics

The seven included studies were published between 1991 and 2024 and were conducted in the United Kingdom, the United States, and Italy (Table 1). Four were prospective cohorts, two were cross-sectional, and one combined invasive hemodynamic monitoring with echocardiography. Sample sizes ranged from 11 to 252 patients. All seven scored 7 or higher on the Newcastle-Ottawa Scale. Six of the seven were conducted at dedicated HCM referral centers. The three Gilligan studies from the 1990s originated from a single United Kingdom center and provide the only invasive hemodynamic data in this literature.

The studies differed substantially in how postprandial assessment was performed (Table 2). Two used a standardized 740 kcal mixed meal; the remainder used meals of variable or unspecified composition. Assessment timing ranged from 15 to 60 min after ingestion, with most studies measuring within the 30 to 60 min window. Patient positioning evolved over time: the earlier studies assessed patients supine, Feiner et al. [13] introduced upright standing with exercise, and Massera et al. incorporated treadmill stress echocardiography.

**Table 1:** Study characteristics and quality assessment

Study	Year	Design	Country	*N*	Age (yrs)	Male (%)	NOS	Quality
Gilligan et al.	1991	Prospective cohort	UK	11	47 ± 15	73	8/9	High
Gilligan et al.	1996a	Cross-sectional	UK	20*	48 ± 14	70	8/9	High
Gilligan et al.	1996b	Prospective cohort	UK	70	45 ± 15	63	7/9	High
Feiner et al.	2013	Prospective cohort	USA	20	NR	NR	7/9	High
Adams et al.	2015	Cross-sectional	USA	45†	53 ± 16	65	8/9	High
La Canna et al.	2020	Prospective cohort	Italy	92	54 ± 16	61	9/9	High
Massera et al.	2024	Prospective cohort	USA	252	58 ± 16	61	9/9	High

NOS = Newcastle-Ottawa Scale; NR = not reported. *Gilligan 1996a enrolled 20 HCM patients (10 with and 10 without postprandial symptoms) and 10 healthy controls; the controls are not included in the analysis. †Adams et al. enrolled 45 HCM patients and 10 healthy controls; the controls are not included in the patient count

**Table 2:** Meal protocols and assessment methods

Study	Meal	Calories	Timing After Meal	Position	Postprandial Assessment Performed
Gilligan 1991	Standardized mixed	740 kcal	45 min	Supine	Rest and exercise, with invasive hemodynamics
Gilligan 1996a	Standardized mixed	740 kcal	15–60 min	Supine	Rest
Gilligan 1996b	Not specified	NR	NR	NR	Rest
Feiner et al.	Patient-selected, commercial	NR	60 min	Standing	Exercise (standing post-exercise)
Adams et al.	Standardized mixed	NR	30 min or symptom onset	Supine	Rest
La Canna et al.	Standardized midday	NR	30–60 min	Supine	Rest (Valsalva and exercise as fasting comparators)
Massera et al.	Patient-directed	NR	30–45 min	Rest supine, exercise upright	Rest, provocation, and treadmill stress

NR = not reported. The final column indicates whether postprandial measurement was made at rest, with provocation, with exercise, or in combination

### LVOT gradient changes with postprandial assessment

Across all seven studies, postprandial assessment produced higher LVOT gradients than fasting measurement, although the magnitude varied with the population studied and the method used. Table 3 summarizes the five studies that reported quantitative gradient data. The two remaining Gilligan studies reported LVOT velocity rather than pressure gradients.

La Canna et al. [14] enrolled 92 patients with no resting obstruction and measured the LVOT gradient under fasting, postprandial, and provoked conditions. Postprandial assessment identified a gradient of 30 mmHg or greater in 68 of 92 patients (74%), compared with 23% by Valsalva maneuver and 33% by exercise (*p* < 0.001).

Massera et al. [12] reported the largest cohort, 252 patients without known obstruction who underwent postprandial resting and treadmill stress echocardiography after a non-standardized meal. The median resting LVOT gradient rose from 0 mmHg fasting to 9.0 mmHg postprandially (*p* < 0.0001). Among patients who did not reach the 50 mmHg threshold under routine conditions (49 patients, 19.4%, reached it routinely), an additional 90 patients (35.7%) reached it with postprandial assessment, and 38 patients (15.1%) reached it only with postprandial stress echocardiography.

Feiner et al. [13] applied postprandial standing exercise echocardiography to 20 patients who had already tested negative on conventional fasting exercise testing. Thirteen of 20 (65%) developed significant obstruction, with the median gradient rising from 25 mmHg fasting to 61 mmHg after postprandial exercise (*p* = 0.03).

Adams et al. [15] observed smaller changes after a standardized meal. The mean gradient increased by 5.0 ± 8.3 mmHg in symptomatic patients, compared with 0.7 ± 1.1 mmHg in controls. The difference between symptomatic and asymptomatic HCM patients was not statistically significant. Gilligan et al. (1996a) similarly found a postprandial increase in LVOT velocity in both symptomatic and asymptomatic patients (*p* < 0.05 in both), with greater absolute velocities in the symptomatic group.

**Table 3:** Quantitative LVOT gradient results

Study	Population	Method	Fasting	Postprandial	Significance
La Canna et al.	92, no resting obstruction	Rest echo; Valsalva and exercise comparators	No resting obstruction	68/92 (74%) ≥ 30 mmHg	vs. Valsalva 23%, exercise 33%; *p* < 0.001
Massera et al.	252, without known obstruction	Rest + treadmill stress; non-standardized meal	0 [0–14.0] mmHg	9.0 [0–38.0] mmHg (rest)	*p* < 0.0001
Adams et al.	18 symptomatic, 27 asymptomatic	Rest echo; standardized meal	23.4 ± 17.6 (symptomatic); 25.1 ± 33.1 (asymptomatic)	+ 5.0 ± 8.3 mmHg change (symptomatic)	vs. controls + 0.7 ± 1.1; symptomatic vs. asymptomatic NS
Feiner et al.	20, negative on conventional testing	Standing post-meal exercise	25 [17–37] mmHg	61 [20–85] mmHg	13/20 (65%); *p* = 0.03
Gilligan 1996a	10 symptomatic, 10 asymptomatic	LVOT velocity by Doppler	2.8 ± 1.3 m/s (symptomatic); 1.8 ± 1.3 (asymptomatic)	3.6 ± 1.5 (symptomatic); 2.4 ± 1.3 (asymptomatic)	*p* < 0.05 both groups

Data are mean ± SD or median [IQR]. NS = not significant. Feiner et al. enrolled patients pre-selected for negative conventional testing, which limits direct comparison with the other cohorts

### Comparison with conventional provocative maneuvers

Three studies allowed direct comparison of postprandial assessment with conventional provocative testing (Table 4). La Canna et al. [14] provided the most rigorous comparison, with postprandial assessment identifying obstruction in 74% of patients versus 23% by Valsalva and 33% by exercise in the same patients (*p* < 0.001). Massera et al. [12] found that postprandial testing identified an additional 35.7% of patients reaching the 50 mmHg threshold beyond routine assessment. Feiner et al. [13] reported a 65% detection rate, although this cohort was selected specifically for prior negative conventional testing, so its detection rate cannot be compared directly with the other two.

**Table 4:** Diagnostic yield versus conventional provocative testing

Study	*N*	Postprandial Detection	Conventional Testing	Comparison
La Canna et al.	92, no resting obstruction	68/92 (74%) ≥ 30 mmHg	Valsalva 23%; exercise 33%	*p* < 0.001 vs. both
Massera et al.	252, without known obstruction	+ 90 (35.7%) reached ≥ 50 mmHg	49 (19.4%) reached ≥ 50 mmHg routinely	*p* < 0.0001
Feiner et al.	20, negative on conventional testing	13/20 (65%)	Negative by inclusion	*p* = 0.03; selected population

Feiner et al. was enriched for negative conventional testing; its detection rate is not directly comparable to the other studies

### Hemodynamic mechanisms

Gilligan et al. (1991) provided the only invasive hemodynamic data, studying 11 HCM patients before and 45 min after a standardized 740 kcal meal (Table 5). The systemic vascular resistance index fell by 17 ± 14%, while mean right atrial pressure rose by 31 ± 21%, mean pulmonary artery pressure by 14 ± 14%, and pulmonary capillary wedge pressure by 17 ± 14% (all *p* < 0.05). The cardiac index rose by 18 ± 10%, driven by an increase in heart rate without a significant change in stroke volume.

**Table 5:** Invasive hemodynamic changes with postprandial assessment (Gilligan et al. 1991, *n* = 11)

Parameter	Change From Fasting	Significance
Systemic vascular resistance index	−17 ± 14%	*p* < 0.05
Mean right atrial pressure	+ 31 ± 21%	*p* < 0.05
Mean pulmonary artery pressure	+ 14 ± 14%	*p* < 0.05
Pulmonary capillary wedge pressure	+ 17 ± 14%	*p* < 0.05
Cardiac index	+ 18 ± 10%	*p* < 0.05

Measured 45 min after a 740 kcal meal. The increase in cardiac index was driven by a higher heart rate without a significant change in stroke volume

### Clinical correlates and treatment

Three studies reported on the clinical relevance of postprandial obstruction (Table 6). Gilligan et al. [16] found that over one-third of 70 HCM patients reported postprandial symptom exacerbation and that these patients were more symptomatic, with greater angina frequency and reduced exercise capacity. Feiner et al. [13] reported that 12 of 13 patients with postprandial obstruction (92%) had attributable symptoms; 9 received targeted therapy with beta-blockers, disopyramide, or myectomy, and quality-of-life scores improved from 29 ± 19 to 12 ± 19 (*p* < 0.02). Among the 71 patients treated in the Massera cohort, 32 (45.1%) had obstruction of 50 mmHg or greater identified only after eating, meaning that nearly half of treated patients would have been classified as having no obstruction on conventional assessment alone.

**Table 6:** Clinical correlates and treatment

Study	Postprandial Symptoms	Treatment	Outcome
Gilligan 1996b	Over one-third	NR	Patients with postprandial symptoms were more symptomatic, with greater angina frequency and reduced exercise capacity
Feiner et al.	12/13 (92%) with obstruction had symptoms	9/13 treated (beta-blocker, disopyramide, or myectomy)	Quality of life improved: 29 ± 19 → 12 ± 19 (*p* < 0.02)
Massera et al.	NR	71 treated; 32/71 (45.1%) had ≥ 50 mmHg only after eating	Symptom improvement reported; no long-term data

NR = not reported. Treatment outcomes are from short-term follow-up only; no included study reported long-term outcomes

## Discussion

We found that across seven studies spanning three decades, postprandial assessment provoked clinically significant LVOT obstruction in a substantial proportion of HCM patients who appeared to have no obstruction on conventional evaluation. The effect was consistent despite wide variation in meal protocols and assessment methods. In the current treatment era, where cardiac myosin inhibitors are approved for symptomatic obstructive HCM and septal reduction therapy requires documented gradients, the distinction between a patient who reaches 50 mmHg only after eating and one classified as having no obstruction has direct consequences for treatment eligibility.

The strongest comparative evidence comes from La Canna et al. [14], who measured the LVOT gradient under fasting, postprandial, and provoked conditions in the same 92 patients. Postprandial assessment identified obstruction in 74% of patients, compared with 23% by Valsalva, the maneuver most often used in practice. Massera et al. [12] found a similar pattern in a larger and less selected cohort: more than a third of patients reached the 50 mmHg threshold only after eating, and nearly half of all treated patients in that cohort had their obstruction identified exclusively by postprandial testing. On conventional assessment alone, these patients would have been considered to have no obstruction and managed accordingly.

The hemodynamic data from Gilligan et al. explain why postprandial assessment may outperform the Valsalva maneuver. Eating reduces systemic vascular resistance through splanchnic vasodilation and raises cardiac filling pressures, while heart rate increases without a corresponding rise in stroke volume. This combination lowers afterload and shortens diastolic filling, conditions that favor systolic anterior motion and dynamic obstruction. The Valsalva maneuver produces a transient reduction in preload that depends heavily on patient effort and examiner technique, whereas the postprandial state is a sustained physiologic change that more closely reproduces the conditions under which patients become symptomatic.

The heterogeneity of meal protocols across studies has a bearing on clinical translation. Significant postprandial obstruction was provoked using both standardized 740 kcal meals and non-standardized patient-directed eating, which suggests that the effect does not depend on precise meal composition. This is reassuring for clinical use, since a test that required a tightly controlled meal would be difficult to reproduce outside a research setting. At the same time, the absence of a standardized protocol limits direct comparison between studies and means that no single meal composition, caloric threshold, or assessment time can yet be recommended.

These findings are relevant to current HCM pharmacotherapy. Cardiac myosin inhibitors reduce LVOT gradients and improve symptoms in obstructive HCM, but they are indicated only when obstruction is documented. A patient with postprandial obstruction that is missed on conventional testing may be denied a therapy from which they would benefit. Postprandial assessment is non-invasive and uses the same echocardiographic technique as standard LVOT measurement. It adds little to the cost of an existing study, although it extends the examination by 30 to 60 min.

Several limitations of the evidence base should be acknowledged. Most studies were conducted at dedicated HCM referral centers by sonographers experienced in upright and post-exercise imaging, and the results may not transfer directly to less specialized settings. The included studies were small, with the exception of the Massera cohort, and the Feiner cohort was selected for prior negative testing, which inflates its apparent yield relative to an unselected population. No study reported long-term outcomes, so whether patients identified by postprandial testing alone have the same prognosis and treatment response as those with conventionally detected obstruction remains unknown. The predominance of positive findings in a small literature also raises the possibility of publication bias.

Several questions remain. Standardized multicenter validation is needed to establish reproducible detection rates outside specialized centers. Long-term outcome data would show whether patients identified by postprandial testing alone have a prognosis and treatment response comparable to those with conventionally detected obstruction. It is also unclear where postprandial assessment belongs in the diagnostic sequence, in particular whether it should follow a negative conventional evaluation or be used earlier in symptomatic patients. Finally, whether treatment response to myosin inhibitor therapy differs between patients identified by postprandial versus conventional assessment is unknown and worth investigating.

## Conclusions

Postprandial assessment identifies LVOT obstruction that conventional provocative maneuvers miss, a finding that is consistent across seven studies conducted over three decades. Because documentation of obstruction now determines eligibility for cardiac myosin inhibitor therapy and septal reduction, this diagnostic gap has direct clinical consequences. The available evidence supports consideration of postprandial testing in symptomatic HCM patients who have no obstruction on conventional evaluation, particularly those who report symptoms after meals. The remaining questions are how the test should be performed and which patients it should be used in, and these are best answered by standardized multicenter study.

## Data Availability

No datasets were generated or analysed during the current study.

## Declarations

### Use of artificial intelligence

The authors used an AI-based language tool (Claude, Anthropic) to assist with language editing and formatting during manuscript revision. The tool was not used for the literature search, study selection, data extraction, or data analysis, all of which were performed by the authors. The authors reviewed and verified all content, including all data, citations, and statistical values, and take full responsibility for the integrity and accuracy of the manuscript.
